# High-throughput analysis of polyethoxylated tallow amine homologs in citrus using a modified QuEChERS-HILIC-MS method

**DOI:** 10.3389/fnut.2022.1061195

**Published:** 2022-11-30

**Authors:** Minjie Li, Hongping Wang, Simeng Li, Xueying Chen, Maojun Jin, Hua Shao, Jing Wang, Fen Jin

**Affiliations:** Key Laboratory of Agro-Product Quality and Safety, Institute of Quality Standards and Testing Technology for Agro-Products, Chinese Academy of Agricultural Sciences, Beijing, China

**Keywords:** pesticide adjuvant, polyethoxylated tallow amine, citrus, QuEChERS, ultrahigh-performance liquid chromatography-mass spectrometry

## Abstract

A new method is described based on ultrahigh-performance liquid chromatography-mass spectrometry (UHPLC) with electrospray mass spectrometry detection for comprehensive quantitative analysis of 66 polyethoxylated tallow amine (POE-tallowamine) homologs in citrus. Efficient separation, reduced band broadening, and high sensitivity were achieved by employing an acetonitrile-aqueous solution containing a 10 mM ammonium formate gradient on a hydrophilic interaction chromatography (HILIC) column with a modified QuEChERS (quick, easy, cheap, effective, rugged, and safe) method. The quantitative accuracy and precision of the method were improved by the use of matrix-matched calibration standards. At spiked levels of (50 + 250) μg/kg, (200 + 1000) μg/kg, and (500 + 2500) μg/kg POE-5 and POE-15 (1:5), the average recoveries of the POE-tallowamine homologs ranged from 71.9 to 112%, with RSDs < 16.6%. The limits of detection (LODs) and limits of quantification (LOQs) for POE-tallowamine homologs were 0.01–2.57 and 0.03–8.58 μg/kg, respectively. The method was successfully applied to determine POE-tallowamine in citrus samples from typical Chinese regions in 2021. POE-tallowamine was detected in all 54 samples, and the highest concentration (143 μg/kg) of POE-tallowamine was found in Jelly orange from Zhejiang Province, which might indicate a higher usage and demand of glyphosate herbicides in Zhejiang.

## Introduction

Polyethoxylated tallow amine (POE-tallowamine) is a class of synthetic surfactants widely used in a variety of pesticides, especially in glyphosate-based herbicide formulations (1–3). POE-tallowamine can improve the plant coverage and penetration of glyphosate through surface tissues (4). However, POE-tallowamine has been demonstrated to have higher toxicity than its active substance glyphosate in a great number of studies (2, 5–7). Considering the concerns about the toxicity of POE-tallowamine and its potential adverse effects on human health, POE-tallowamine has been listed as a co-formulant that is not accepted for inclusion in plant protection products by the European Union (EU) (8). The US Environmental Protection Agency (EPA) and Canada have recently limited the additive dose of POE-tallowamine to less than 25 and 20% in pesticide formulations, respectively (9, 10). The increased attention focused on the occurrence of POE-tallowamine in agroecosystems necessitates the development of sensitive and robust analytical methods for determining these compounds in complex agroproduct matrices.

A typical POE-tallowamine comprises one linear aliphatic chain and two polyethoxylate chains attached to a single nitrogen atom with a range of 2–23 ethoxylate units (EO, *n* = 2–23, Supplementary Figure 1). The linear aliphatic chain is mainly derived from saturated palmitic acid (C16:0), saturated stearic acid (C18:0), and unsaturated oleic acid (C18:1, ω−9). The EO numbers in two ethoxylate chains of POE-tallowamine may equal or differ, rendering POE-tallowamine analysis a major challenge. Reversed-phase liquid chromatography coupled with mass spectrometry (LC-MS) is suitable for the determination of POE-tallowamine as a mixture (11–16). Wang et al. (13) and Rodriguez-Gil et al. (17) determined the total concentrations of a POE-tallowamine mixture in soil by a C18 column based on carbon chain length while lacking single ethoxylated POE-tallowamine homolog chromatographic separation and quantitation. The coelution of ethoxylated POE-tallowamine homologs will lead to competitive ionization suppression during the electrospray process and isobaric interferences between single- and double-charged POE-tallowamine ethoxymers (18–21). Recently, hydrophilic interaction chromatography (HILIC) combined with mass spectrometry was used widely for the separation of polydisperse surfactants in petrochemicals that are difficult to separate in reversed-phase chromatography (19, 22–26). To the best of our knowledge, there are no reports of useful techniques for the determination of single ethoxylated POE-tallowamine in aro-product samples.

In this study, we developed a QuEChER-HILIC-MS method for quantification of trace levels of individual POE-tallowamine homologs in citrus samples. A matrix effect study was conducted, demonstrating that ion suppression from sample matrices was decreased. This method was then applied to characterize the distribution of different POE-tallowamine homologs in citrus samples, providing more foundation information for human health risk assessment.

## Materials and methods

### Chemicals and reagents

POE-2 tallow amine, POE-5 tallow amine, and POE-15 tallow amine (a mixture of POE-tallowamine with an average of 2, 5, and 15 EO units, respectively) were purchased from Chem Service Inc. (West Chester, PA, USA). Acetonitrile (ACN, HPLC grade) and ammonium formate (HPLC grade) were acquired from Thermo Fisher Scientific (Waltham, MA, USA). Ultrapure water (≥18.2 MΩ⋅cm) was generated by the Milli-Q purification system (Millipore, Bedford, MA, USA). Primary secondary amine (PSA), octadecyl (C18) sorbents, and graphitized carbon black (GCB) were supplied by Agela Technologies (Tianjin, China). Sodium chloride and anhydrous magnesium sulfate of analytical grade were obtained from Sinopharm Chemical Reagent Company (Beijing, China).

Stock standard solutions of POE-2, POE-5, and POE-15 (10.0 g/L) were prepared in ACN, and the spiked solutions were a mixture of POE-5 and POE-15 (1:5). The working solutions were achieved by appropriate dilution with ACN. All of the solutions were stored at −20°C. According to Tush et al. (15), Cz(s/u) EOn was used to describe the individual homologs, where *z* is the number of carbon atoms, *s* is a saturated tallow moiety, *u* is a monounsaturated tallow moiety, and *n* is the total number of EO units from two ethoxylate chains. It was assumed that C16s, C18s, and C18u accounted for 90% of the mixtures, and individual homologs obtained an equal instrument response, which was consistent with the analysis of nonylphenol ethoxylates (NPEOs) (27).

### Sample collection and preparation

Fifty-four ripe citrus samples were collected from the main citrus production regions in China, including Guangxi province, Zhejiang province, Hunan province, and Chongqing municipality, which contained Shatang mandarin, Jelly orange, Bingtang sweet orange, Satsuma mandarin, Orah citrus, and W. Murcott Tangerine. Six to twelve parallel samples were collected for each species from different regions. All the samples were homogenized and stored at −20°C before analysis.

Frozen citrus samples were allowed to thaw completely before being thoroughly mixed and subsampled for extraction. After thawing at room temperature, 10 g homogenized citrus samples were extracted with 10 mL of 1% formic acid in acetonitrile in a 50-mL polypropylene centrifuge tube. Each sample was vortexed for 1 min with a Vortex-Genie 2 (Scientific Industries Inc., Bohemia, NY, USA) and ultrasonically extracted by an ultrasonic cleaner (Model KQ-500DB, Kunshan Ultrasonic Instrument Co., Ltd., Jiangsu, China) at room temperature for 20 min. Subsequently, 4 g of anhydrous magnesium sulfate and 1 g of sodium chloride were added to the centrifuge tube and vortexed for 1 min. After centrifugation at 5,000 rpm for 10 min, 1 mL of the supernatant was pipetted into a 2.5-mL clean centrifuge tube that contained 50 mg PSA and 5 mg GCB, vortexed and centrifuged at 10,000 rpm for 5 min. Finally, the supernatant was filtered through a 0.22-μm membrane (Bonna-Agela Technologies Inc., Tianjin, China) and transferred into a 1.5-mL autosampler vial for direct analysis by ultrahigh-performance liquid chromatography-mass spectrometry (UHPLC–MS).

### Instrumental conditions

Quantitative analyses of POE-tallowamine were conducted by a Shimadzu Triple Quadrupole LCMS–8050 system (Shimadzu, Kyoto, Japan) in positive electrospray ionization (ESI) mode. A Waters CQUITY UPLC^®^ BEH HILIC column (2.1 × 100 mm, 1.7 μm particle size) was used to separate the analytes. A gradient procedure was performed using mobile phases A (water containing 10 mM ammonium formate) and B (ACN) at a flow rate of 0.30 mL/min. The injection volume was 5 μL, and the column temperature was maintained at 25°C. The gradient elution program was as follows: mobile phase B was initiated with 92% (held for 1 min), followed by a linear decrease to 80% in 10 min and kept for 2 min. Then, it was increased to 92% to maintain the initial chromatographic condition within 4 min. The back pressure was 16.6 MPa. The column temperature was maintained at 25°C. MS detection was carried out using a time-programmed selected ion monitoring mode with an LCMS-8050 tandem quadrupole mass spectrometer (Shimadzu, Kyoto, Japan). The ion source temperature was 450°C. The identification of target compounds was based on mass measurement of different adducts, namely, [M + H]^+^ and [M + H + NH_4_]^2+^. System control, data acquisition, and data analyses were performed with LabSolutions software (version 5.82, Shimadzu).

## Results and discussion

### Optimization of spectrometry and chromatography conditions

**FIGURE 1:**
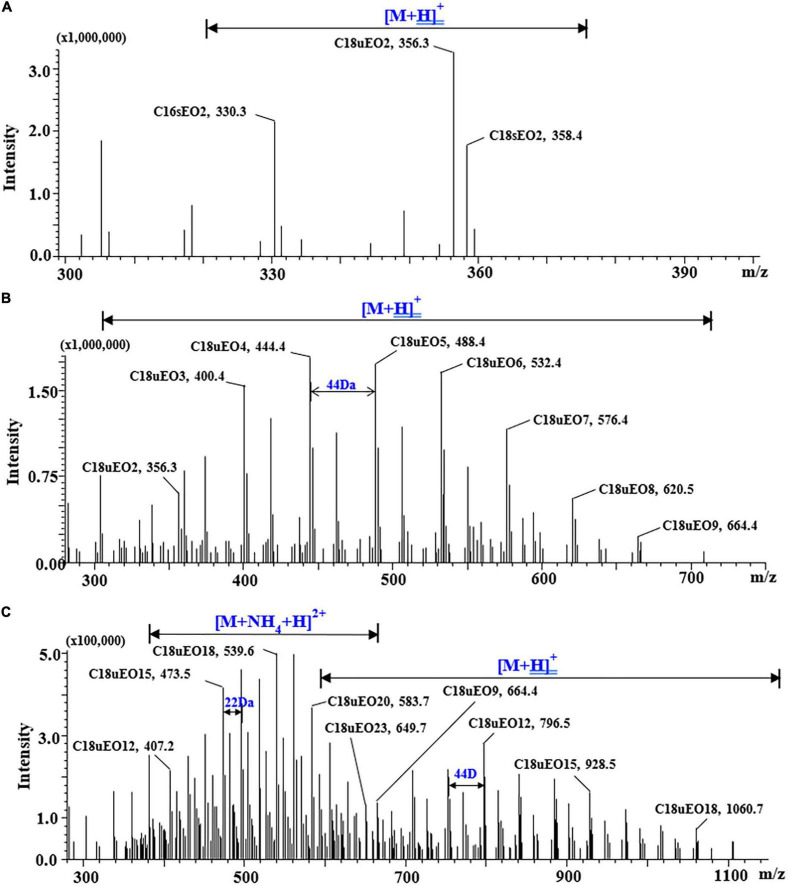
Mass spectrum of standard polyethoxylated tallow amine (POE-tallowamine) by using ultrahigh-performance liquid chromatography-mass spectrometry (UHPLC-MS). **(A)** POE-2 (0.5 mg/L), **(B)** POE-5 (1 mg/L), and **(C)** POE-15 (5 mg/L).

A typical POE-tallowamine is subclassified based on the average ethoxylate lengths into POE-2, POE-5, and POE-15. As shown in Figures 1A,B, the single-charged adduct ions ([M + H]^+^) were observed in POE-2 (*n* = 2) and POE-5 (*n* = 2–9) with mass differences of 44 Da between neighboring homologs. This observation was consistent with the results of previous work (15). For POE-15 (*n* = 12–20), except for [M + H]^+^, double-charged adduct ions ([M + H + NH_4_]^2+^) were simultaneously detected with mass differences of 22 Da between neighboring homologs (Figure 1C). We found that the [M + H + NH_4_]^2+^ adducts exhibited stronger responses than the [M + H]^+^ adducts when POE-15 homologs had more EO units (*n* > 13). For example, the responses of double-charged adducts ([M + H + NH_4_]^2+^) provided by C16sEO14, C16sEO15, and C16sEO16 were 1.5-fold, 5-fold, and 9-fold higher than those of the corresponding single-charged adducts ([M + H]^+^) in the chromatograms, respectively (Supplementary Figure 2). This is the first report on the adduct ions ([M + H + NH_4_]^2+^) of POE-tallowamine. Similar phenomena were found in NPEOs and tristyrylphenol ethoxylate (TSPEOs) homologs, where two clusters of characteristic signals (single- and double-charged adduct ions) were chosen as the precursor ion (28, 29). Similarly, the responses of double-charged adducts of NPEOs and TSPEOs also increased with EO chain length. Therefore, [M + H]^+^ adducts were chosen as base ions of POE-tallowamine homologs (*n* = 2–13), whereas [M + H + NH_4_]^2+^ adducts were base ions for POE-tallowamine homologs (*n* = 14–23) (Supplementary Table 1).

**FIGURE 2:**
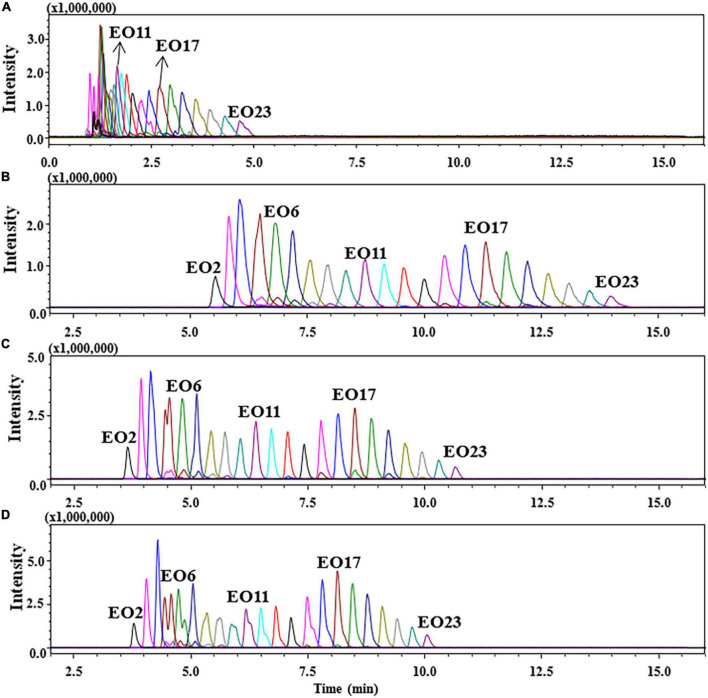
Chromatograms of C16s spiked at POE-5 (1 mg/L) and POE-15 (5 mg/L) in different aqueous phases. **(A)** 0.2% formic acid in water, **(B)** 1 mM ammonium formate in water, **(C)** 10 mM ammonium formate in water, and **(D)** 2% formic acid and 10 mM ammonium formate in water.

Considering that the unfavorable separation and coelution of POE-tallowamine by reversed-phase chromatography column in previous studies (11–16). POE-tallowamine was separated based on its hydrophilic moiety by a HILIC column. One of the main advantages of HILIC is the efficient separation of polar compounds in aqueous-organic mobile phases rich in organic solvents (usually ACN) (30). In general, the pH value and additives of the mobile phase have a significant impact on retention and selectivity in the chromatographic column (31–33). Figure 2 presents the chromatograms of standard mixtures containing 1 mg/L POE-5 and 5 mg/L POE-15 at different percentages of formic acid and ammonium formate. As exemplified by C16s in Figure 2A, the chromatographic peaks of C16s were not well separated between 1 and 6 min when the aqueous phase consisting of only formic acid was used, which displayed a reduction in retention and deterioration in peak shape. To improve the retention selectivity and peak profiles, the addition of ammonium formate to the mobile phase was also investigated. As shown in Figures 2B,C, the chromatographic peak shape was greatly improved, and the retention time was moved forward for POE-tallowamine with the increase in the ammonium formate concentration from 1 to 10 mM. The reason could be that salt could significantly affect the adsorption behavior of the stationary phase (30, 34). Compared to 1 mM ammonium acetate, sharper, and symmetrical peak shapes were observed by using a concentration of 10 mM ammonium acetate, with the band broadening reducing approximately 42–50% and the sensitivity increasing one-fold. This result suggested that the addition of ammonium formate also helped to the LC-electrolyte effects of POE-tallowamine. Similar effects of ammonium formate were reported in the separation of saccharide and antibiotics (34, 35). Moreover, the effect of the addition of 0.2% formic acid and 10 mM ammonium formate was also evaluated (Figure 2D). The peak shapes of C16sEO6, C16sEO10, C16sEO11, and C16sEO15 were distinctly split, and C16sEO4-7 overlapped obviously, negatively impacting the quantitation of the target analytes. Consequently, 10 mM ammonium formate added to the mobile phase was ultimately selected in our study, providing optimum analytical separation.

### Optimization of the sample preparation

**FIGURE 3:**
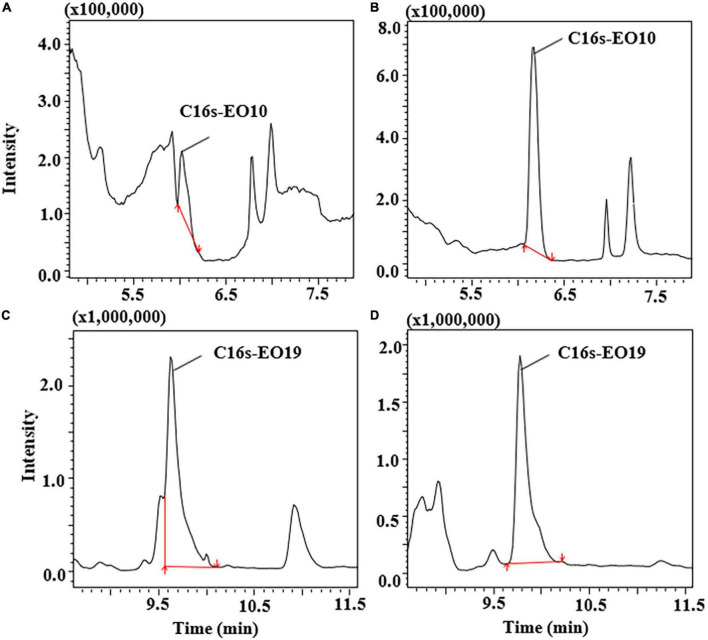
The ion chromatograms of C16s-EO10 and C16s-EO19 in different solvent extraction performances. **(A)** C16s-EO10 extracted by 1.5% formic acid in acetonitrile, **(B)** C16s-EO10 extracted by 1% formic acid in acetonitrile, **(C)** C16s-EO19 extracted by 0.1% formic acid in acetonitrile, and **(D)** C16s-EO19 extracted by 1% formic acid in acetonitrile. The spiked levels were 0.2 mg/L POE-5 and 1 mg/L POE-15.

ACN and ACN with different degrees of acidification were tested to optimize the extraction procedure, including 0.1, 0.5, 1, and 1.5% formic acid (FA) in ACN. As shown in Supplementary Figure 3, the recoveries of different extraction solvents for POE-tallowamine homologs were in the range of 63.1–106%. However, the recoveries of the compounds showed improvement upon addition of 0.1–1.5% FA, which may be because the use of acidification during the extraction could improve the extraction efficiency of cationic surfactants (36, 37). Satisfactory recoveries of antibiotics were also achieved by adding formic acid to adjust the pH (35). In comparison with C16sEO10 extracted by 1.5% FA in ACN (Figure 3A). Figure 3B shows obvious separation of C16sEO10 from impurity peaks with a good peak shape when extracted by 1% FA in ACN. The resolution of C16sEO10 was significantly increased by four-fold under 1% FA in ACN. As shown in Figures 3C,D, an adverse separation between C16sEO19 and impurity peaks under 0.1% FA in ACN and a refined separation of C16sEO19 under 1% FA in ACN were clearly observed. This might affect the hydrophobic interactions of the compounds as a result of their pH, leading to a better separation of POE-tallowamine homologs. Therefore, 1% FA in ACN treatment was ultimately selected as the extraction solvent.

**FIGURE 4:**
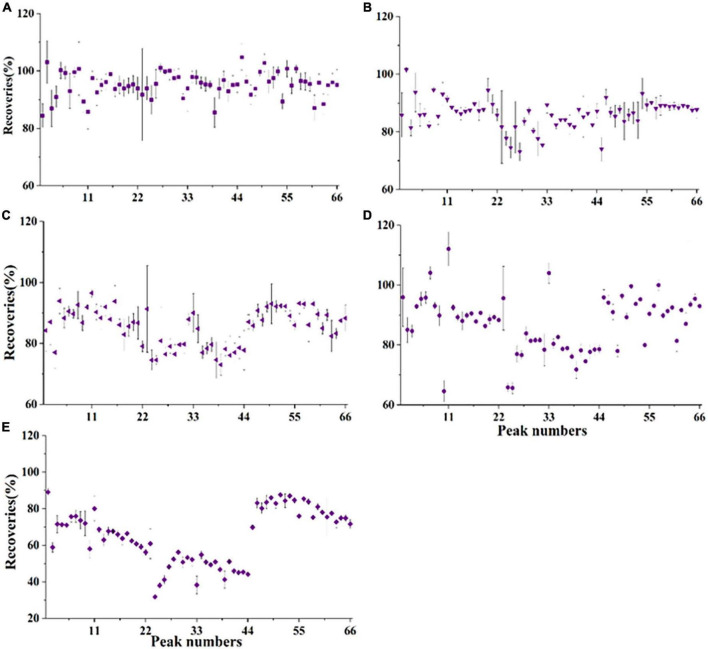
Recoveries of different dispersive solid-phase extraction (DSPE) sorbents for polyethoxylated tallow amine (POE-tallowamine) homologs in the matrix (*n* = 3). The spiked levels were 0.2 mg/L POE-5 and 1 mg/L POE-15. **(A)** 50 mg PSA, **(B)** 50 mg C18, **(C)** 10 mg GCB, **(D)** 50 mg PSA + 5 mg GCB, and **(E)** 50 mg PSA + 10 mg GCB. The peak numbers are the same as in Supplementary Table 1.

To obtain high sensitivities and lower method limits, the dispersive solid-phase extraction (DSPE) method was used to purify the acetonitrile phase. Different DSPE sorbents were tested during optimization in this study. First, 50 mg PSA, 50 mg C18, and 10 mg GCB were evaluated by purifying crude citrus sample extracts spiked with POE-5 (200 μg/kg) and POE-15 (1000 μg/kg). As shown in Figures 4A–C, PSA, C18, and GCB showed good recoveries for all 66 POE-tallowamine homologs (PSA: 84.4–105%, C18: 73.1–102%, and GCB: 72.8–96.6%). The difference is that the analytical sensitivities for PSA were approximately four-fold higher than those for C18, as exemplified by C16sEO10 (Supplementary Figure 4). This might be because PSA has a greater capacity than aminopropyl sorbent based on the extra secondary amino group and provides adsorption properties for fatty acids (38–40). Additionally, previous studies indicated that GCB was highly effective in reducing coextracted pigments in QuEChERS (39, 41). Thus, different combinations of PSA and GCB sorbents were further optimized to improve adsorption effects. With the increase in GCB from 5 to 10 mg, the recoveries were observed to decrease from 64.6–112 to 31.8–89.1% (Figures 4D,E). Therefore, 50 mg PSA and 5 mg GCB were selected as the DSPE sorbent for all POE-tallowamine homologs in this study.

### Method validation

To confirm the practicability of the present method, matrix effects were evaluated for POE-tallowamine by using the slope ratio of matrix-matched standard curves with a solvent standard calibration curve. Matrix-matched and solvent standard curves were created for POE-tallowamine with different spiked concentrations in the range of 5–1,000 ng/mL POE-5 and 25–5,000 ng/mL POE-15. As shown in Supplementary Figure 5, POE-tallowamine homologs in the tested citrus exhibited matrix suppression effects. Therefore, matrix-matched calibration curves were built for the POE-tallowamine analysis to reduce the influence of the matrix.

The recovery experiment was conducted to evaluate the method accuracy by spiking at three times the original concentration of the standard mixture into citrus samples. As shown in Table 1, at spiked levels of (50 + 250) μg/kg, (200 + 1,000) μg/kg, and (500 + 2,500) μg/kg POE-5 and POE-15 (1:5), the relative recoveries ranged from 71.9 to 112% with a relative standard deviation (RSD) < 16.6% in citrus samples. The correlation coefficients (R^2^) of determination were typically greater than 0.9900, which indicated that linearity was acceptable for all target compounds over the citrus-relevant concentration range. Moreover, the limits of detection (LODs) and quantification (LOQs) of the POE-tallowamine homologs were estimated by analyzing spiked samples at low concentrations. LODs and LOQs were calculated on the basis of peak-peak signal-to-noise (S/N) values of 3 and 10, respectively (42–44). The obtained LODs and LOQs of the POE-tallowamine homologs were in the range of 0.01–2.57 and 0.03–8.58 μg/kg for citrus samples, respectively. These results demonstrated that the present QuEChERS-HILIC-MS method was suitable for the determination of POE-tallowamine homologs from various citrus matrices. It has been reported that a maximum of 15 homologs of C16s, C18s, and C18u were detected in previous analytical studies (12, 14). However, the proposed method allowed us to simultaneously and selectively determine 66 POE-tallowamine homologs, significantly improving the high-throughput analysis of POE-tallowamine.

**TABLE 1:** Method validation of polyethoxylated tallow amine (POE-tallowamine) in citrus (*n* = 3).

Compound	1st spiked level (μg/kg)	Average recovery 1 (%)	RSD 1 (%)	2nd spiked level (μg/kg)	Average recovery 2 (%)	RSD 2 (%)	3rd spiked level (μg/kg)	Average recovery 3 (%)	RSD 3 (%)	Regression equation	*R* ^2^	LOD (mg/kg)	LOQ (mg/kg)
C16sEO2	0.20	/	/	0.79	110	8.12	1.98	108	13.4	*Y* = 10638.1X + 4.14534e + 006	0.9991	0.09	0.30
C16sEO3	1.38	96.7	7.88	5.54	85.7	2.98	13.8	96.4	1.56	*Y* = 74328.9X + 755,073	0.9998	0.37	1.23
C16sEO4	2.54	108	8.66	10.2	102	4.84	25.4	95.4	0.36	*Y* = 119338X + 2.25222e + 006	0.9999	0.16	0.55
C16sEO5	2.54	98.4	3.91	10.2	92.6	3.48	25.4	99.0	1.51	*Y* = 125945X + 1.051142e + 006	0.9997	0.16	0.53
C16sEO6	3.07	103	0.85	12.3	102	2.43	30.7	99.8	1.65	*Y* = 106278X + 1.16466e + 006	0.9996	0.67	2.22
C16sEO7	3.46	101	1.00	13.9	92.3	2.73	34.6	99.2	1.91	*Y* = 91239X + 65,535.5	1.0000	1.51	5.04
C16sEO8	4.29	97.9	1.39	17.2	96.4	7.41	42.9	95.2	6.68	*Y* = 53491.8X − 486,151	0.9995	1.87	6.25
C16sEO9	5.68	96.6	4.97	22.7	103	3.20	56.8	99.3	2.27	*Y* = 95794.7X + 384,218	0.9998	0.28	0.94
C16sEO10	7.37	84.2	8.38	29.5	103	5.77	73.7	96.8	0.31	*Y* = 13061.6X + 51,512.3	0.9957	0.31	1.02
C16sEO11	8.63	88.3	6.02	34.5	99.7	8.63	86.3	100	2.13	*Y* = 19685.6X − 305,829	0.9929	1.48	4.93
C16sEO12	8.59	104	3.86	34.4	92.9	14.0	85.9	96.8	9.40	*Y* = 53497.6X − 659,003	0.9979	0.77	2.56
C16sEO13	8.66	90.1	11.3	34.6	92.1	6.69	86.6	98.6	6.42	*Y* = 72089.3X + 1.15542e + 007	0.9992	0.52	1.75
C16sEO14	8.03	91.1	6.67	32.1	104	7.87	80.3	102	2.70	*Y* = 47360.7X − 185,358	0.9988	1.24	4.13
C16sEO15	7.24	95.8	2.09	29.0	90.7	4.44	72.4	102	7.32	*Y* = 106205X − 1.25228e + 006	0.9973	1.81	6.02
C16sEO16	5.85	101	2.61	23.4	97.4	4.69	58.5	98.6	2.34	*Y* = 141174X − 1.36994e + 006	0.9984	2.26	7.52
C16sEO17	4.46	104	2.56	17.8	89.4	8.72	44.6	96.5	12.1	*Y* = 150764X + 5.85730e + 006	0.9996	0.24	0.79
C16sEO18	3.17	96.9	2.81	12.7	102	1.95	31.7	91.0	1.12	*Y* = 67337.9X − 1.39652e + 006	0.9962	1.07	3.56
C16sEO19	1.98	94.3	7.36	7.93	93.7	4.62	19.8	94.3	8.88	*Y* = 164513X + 3.33064e + 006	0.9986	0.34	1.14
C16sEO20	1.19	94.8	5.76	4.76	96.7	3.63	11.9	89.8	3.24	*Y* = 153890X − 990,697	0.9996	0.16	0.55
C16sEO21	0.79	97.4	4.35	3.17	95.3	4.45	7.93	101	2.87	*Y* = 123607X − 1.20720e + 006	0.9988	0.06	0.19
C16sEO22	0.40	103	2.86	1.59	96.7	3.66	3.97	97.8	0.16	*Y* = 88312.9X − 785,431	0.9985	0.03	0.11
C16sEO23	0.10	102	1.58	0.40	85.4	11.9	0.99	100	3.29	*Y* = 45964.2X − 407,883	0.9989	0.02	0.06
C18sEO2	0.16	/	/	0.66	108	10.1	1.65	112	12.9	*Y* = 5755.03X + 5.80699e + 006	0.9943	0.12	0.40
C18sEO3	1.02	71.9	16.6	4.09	81.6	5.35	10.2	79.8	2.82	*Y* = 59566.9X + 4.01101e + 006	0.9999	0.52	1.73
C18sEO4	1.62	82.3	4.61	6.46	102	6.90	16.2	91.5	2.06	*Y* = 100730X + 1.24447e + 006	0.9999	0.22	0.72
C18sEO5	1.55	95.4	2.49	6.20	107	2.41	15.5	92.9	2.35	*Y* = 105369X − 331,227	1.0000	0.42	1.40
C18sEO6	2.34	103	3.09	9.37	99.7	3.19	23.4	94.7	2.93	*Y* = 54060.3X + 157,924	0.9996	0.38	1.25
C18sEO7	2.87	93.0	3.21	11.5	91.4	2.78	28.7	96.9	1.56	*Y* = 95244.9X − 1.09864e + 006	0.9997	0.29	0.96
C18sEO8	3.50	80.1	4.92	14.0	90.2	9.27	35.0	95.7	7.50	*Y* = 6427.08X − 20,036.8	0.9992	1.39	4.62
C18sEO9	4.36	104	8.59	17.4	102.5	3.77	43.6	101	3.75	*Y* = 70137X + 1.25458e + 006	0.9998	0.84	2.80
C18sEO10	5.39	101	2.06	21.5	91.1	7.79	53.9	90.7	2.11	*Y* = 63797.9X + 318,786	0.9999	0.24	0.79
C18sEO11	5.88	102	5.47	23.5	103	7.80	58.8	103	4.52	*Y* = 5814.33X − 154,834	0.9947	2.57	8.58
C18sEO12	6.28	87.7	1.51	25.1	90.6	3.33	62.8	91.4	1.14	*Y* = 72061X − 1.15738e + 006	0.9960	0.82	2.75
C18sEO13	6.45	80.1	7.53	25.8	107	9.72	64.5	103	2.00	*Y* = 26495.5X − 85,556.7	0.9978	1.77	5.91
C18sEO14	6.35	94.0	4.03	25.4	92.3	6.32	63.5	94.3	2.63	*Y* = 60607X − 179,269	0.9980	0.35	1.17
C18sEO15	5.75	90.6	0.70	23.0	97.7	5.82	57.5	93.5	1.97	*Y* = 122675X − 770,581	0.9989	1.02	3.40
C18sEO16	4.76	95.3	3.02	19.0	94.4	5.67	47.6	99.0	4.03	*Y* = 137611X − 2.62652e + 006	0.9970	1.39	4.62
C18sEO17	3.37	100	3.24	13.5	88.6	2.29	33.7	99.7	1.58	*Y* = 156033X − 1.17992e + 006	0.9992	0.75	2.52
C18sEO18	2.38	106	5.96	9.52	94.6	7.39	23.8	107	4.67	*Y* = 159386X + 1.32733e + 007	0.9998	0.04	0.13
C18sEO19	1.49	92.0	1.70	5.95	98.4	3.09	14.9	98.1	3.30	*Y* = 146972X − 2.07112e + 006	0.9975	0.07	0.24
C18sEO20	0.99	101	2.97	3.97	86.4	9.75	9.92	96.6	3.92	*Y* = 139825X − 793,609	0.9992	0.02	0.08
C18sEO21	0.59	101	2.42	2.38	94.1	4.59	5.95	101	2.50	*Y* = 108112X − 433,052	0.9990	0.01	0.03
C18sEO22	0.40	96.3	1.71	1.59	90.7	6.84	3.97	102	1.77	*Y* = 80682.9X − 529,815	0.9983	0.02	0.07
C18sEO23	0.20	100	1.98	0.79	92.4	5.82	1.98	103	1.10	*Y* = 54178.9X − 451,563	0.9990	0.04	0.12
C18uEO2	0.36	/	/	1.45	101	3.06	3.63	97.6	1.98	*Y* = 33183.1X + 277,382	0.9998	0.10	0.34
C18uEO3	2.21	102	10.2	8.84	88.8	7.03	22.1	97.0	2.84	*Y* = 126808X + 2.26834e + 006	0.9996	0.42	1.40
C18uEO4	3.30	95.8	5.72	13.2	108	6.28	33.0	99.2	1.95	*Y* = 190378X + 862,045	0.9997	0.23	0.76
C18uEO5	3.03	102	0.58	12.1	96.3	4.91	30.3	98.6	1.88	*Y* = 183825X + 507,157	0.9987	0.56	1.88
C18uEO6	6.27	99.2	3.70	25.1	97.8	4.16	62.7	99.2	1.76	*Y* = 160410X + 1.12737e + 006	0.9997	0.82	2.72
C18uEO7	7.56	94.2	5.56	30.2	97.8	3.64	75.6	99.3	1.60	*Y* = 149304X + 3.82661e + 006	0.9995	2.44	8.13
C18uEO8	8.32	102	5.06	33.3	86.2	11.8	83.2	99.4	2.15	*Y* = 71061.9X + 6,323.82	0.9999	0.75	2.52
C18uEO9	8.76	102	1.93	35.0	104	7.10	87.6	99.2	2.40	*Y* = 136566X + 977,788	0.9988	0.95	3.16
C18uEO10	9.58	101	6.17	38.3	91.4	9.37	95.8	99.2	0.65	*Y* = 53277.3X + 1.03266e + 006	1.0000	0.31	1.05
C18uEO11	9.62	91.2	11.7	38.5	98.7	4.59	96.2	103	3.19	*Y* = 8436.05X − 138,838	0.9972	1.14	3.81
C18uEO12	9.98	96.8	14.7	39.9	92.4	3.06	99.8	85.7	8.20	*Y* = 25968.3X + 297,608	0.9975	1.55	5.16
C18uEO13	9.55	101	0.80	38.2	104	8.04	95.5	100	2.36	*Y* = 45051.5X + 824,902	0.9995	0.62	2.06
C18uEO14	8.83	105	4.57	35.3	103	8.85	88.3	96.8	3.53	*Y* = 74165.5X + 2.53922e + 006	0.9979	0.26	0.85
C18uEO15	7.64	99.8	1.67	30.5	90.9	6.23	76.4	99.6	1.79	*Y* = 166842X − 252,087	0.9995	1.39	4.63
C18uEO16	6.05	99.1	3.06	24.2	97.7	4.41	60.5	101	2.90	*Y* = 177829X − 1.54051e + 006	0.9995	0.77	2.56
C18uEO17	4.46	103	1.84	17.8	94.9	4.18	44.6	101	2.27	*Y* = 195438X + 360,378	0.9994	0.52	1.75
C18uEO18	2.97	98.6	1.03	11.9	94.7	4.94	29.7	101	2.80	*Y* = 215319X + 138,618	0.9984	0.03	0.10
C18uEO19	1.88	101	2.27	7.54	93.5	4.82	18.8	101	2.70	*Y* = 207100X − 947,497	0.9993	0.13	0.42
C18uEO20	1.09	98.4	2.38	4.36	90.5	5.82	10.9	101	3.74	*Y* = 177080X − 1.57976e + 006	0.9996	0.08	0.28
C18uEO21	0.69	98.8	7.16	2.78	96.9	5.11	6.94	94.2	4.47	*Y* = 140553X − 1.25333e + 006	0.9994	0.07	0.23
C18uEO22	0.40	101	2.88	1.59	92.9	4.85	3.97	98.6	4.20	*Y* = 106,050X − 959,152	0.9986	0.01	0.04
C18uEO23	0.10	89.1	4.85	0.40	92.1	6.30	0.99	91.1	1.79	*Y* = 67095X − 1.03891e + 006	0.9978	0.02	0.07

### Concentrations and ethoxymer distribution of POE-tallowamine in citrus samples

**FIGURE 5:**
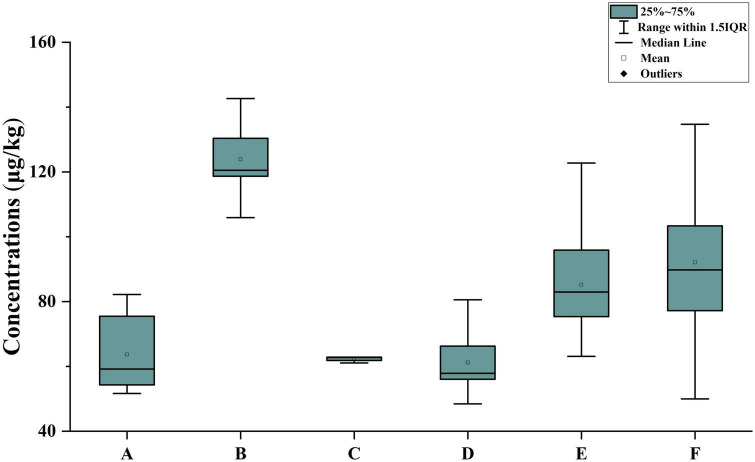
Measured concentrations of ΣPOE-tallowamine in citrus samples from different typical regions. A: Guangxi province, Shatang mandarin, B: Zhejiang province, Jelly orange, C: Hunan province, Bingtang sweet orange, D: Chongqing municipality, Satsuma mandarin, E: Chongqing municipality, Orah citrus, and F: Chongqing municipality, W. Murcott Tangerine.

A total of 54 citrus samples from four main production provinces were analyzed by the method described in this study. As shown in Figure 5, POE-tallowamine homologs were detected in all samples. The total concentrations of POE-tallowamine ranged from 48.5 to 143 μg/kg, and the highest concentration was found in Zhejiang Province. For the six citrus species that we obtained, the highest concentration of POE-tallowamine was found in Jelly orange, with a range of 94.6–143 μg/kg. These results might indicate a higher usage and demand of glyphosate herbicides in Zhejiang Province than in other provinces.

The concentration profiles of POE-tallowamine homologs in citrus samples are shown in Supplementary Figure 6, with a concentration range of 0–59.5 μg/kg. Compared to POE-15 standards, which are widely used in glyphosate-based herbicide formulations (15), the contributions of EO units (*n* = 2–4) in citrus samples increased from 0 to 33.4%. The different distributions of POE-tallowamine homologs were possibly due to different POE-tallowamine technical mixtures applied in agroecosystems (14, 15, 45). Degradation that occurs similar to NPEOs may also contribute to the varying concentration profiles of POE-tallowamine homologs (46, 47). It has been reported that NPEOs with a shorter chain could exhibit stronger toxicity and persistence than those with a longer chain. Therefore, more attention should be given to the toxicity and persistence of EO2-4, which might increase exposure risks to human health and the environment.

## Conclusion

In the present study, we developed a highly sensitive UHPLC-MS method for the simultaneous determination of 66 POE-tallowamine homologs in citrus samples. HILIC achieved efficient separation of POE-tallowamine by a hydrophilic moiety. The validation test of the method demonstrated satisfactory linearity, method detection limit, and precision. This method was successfully applied to analyze typical citrus samples and provided a new reference for the rapid separation and analysis of POE-tallowamine homologs. Based on the developed method, further studies are needed to explore the occurrence and environmental fate of POE-tallowamine.

## Data availability statement

The raw data supporting the conclusions of this article will be made available by the authors, without undue reservation.

## Author contributions

ML: data curation and writing—original draft preparation. HW: writing—reviewing and editing. SL: formal analysis. XC: investigation. MJ: methodology. HS: validation. JW: supervision. FJ: writing—reviewing and editing and funding acquisition. All authors contributed to the article and approved the submitted version.

## References

[1] Lindberg T de Ávila RI Zeller KS Levander F Eriksson D Chawade A An integrated transcriptomic- and proteomic-based approach to evaluate the human skin sensitization potential of glyphosate and its commercial agrochemical formulations. *J Proteomics.* (2020) 217:103647. 10.1016/j.jprot.2020.103647 32006680

[2] Turhan DÖ Güngördü A Ozmen M. Developmental and lethal effects of glyphosate and a glyphosate-based product on *Xenopus laevis* embryos and tadpoles. *Bull Environ Contam Toxicol.* (2020) 104:173–9. 10.1007/s00128-019-02774-z 31932905

[3] Bednářová A Kropf M Krishnan N. The surfactant polyethoxylated tallowamine (POEA) reduces lifespan and inhibits fecundity in *Drosophila melanogaster*- in vivo and in vitro study. *Ecotox Environ Safe.* (2020) 188:109883. 10.1016/j.ecoenv.2019.109883 31704328

[4] Kirkwood RC Hetherington R Reynolds TL Marshall G. Absorption, localisation, translocation and activity of glyphosate in barnyardgrass (*Echinochloa crus-galli* (L) Beauv): influence of herbicide and surfactant concentration. *Pest Manag Sci.* (2000) 56:359–67.

[5] European Food Safety Authority. Request for the evaluation of the toxicological assessment of the co-formulant POE-tallowamine. *EFSA J.* (2015) 13:13. 10.2903/j.efsa.2015.4303 PMC13189434 42169826

[6] Serra L Estienne A Vasseur C Froment P Dupont J. Review: mechanisms of glyphosate and glyphosate-based herbicides action in female and male fertility in humans and animal models. *Cells Basel.* (2021) 10:3079. 10.3390/cells10113079 34831302 PMC8622223

[7] Torres-Badia M Solar-Malaga S Martin-Hidalgo D Hurtado De Llera A Gomez-Candelo A Garcia-Marin LJ Impaired mammalian sperm function and lower phosphorylation signaling caused by the herbicide Roundup^®^ ultra plus are due to its surfactant component. *Theriogenology.* (2021) 172:55–66. 10.1016/j.theriogenology.2021.05.026 34102463

[8] Official Journal of the European Union. *Commission Regulation (EU) 2021/383 of 3 March 2021amending Annex III to Regulation (EC) No 1107/2009 of the European Parliament and of the Council Listing Co-Formulants which are not Accepted for Inclusion in Plant Protection Products.* (2021). Available online at: https://eur-lex.europa.eu/legal-content/EN/TXT/HTML/?uri=CELEX:32021R0383&from=EN (accessed September 30, 2022).

[9] Electronic Code of Federal Regulations. *§180.930 Inert Ingredients Applied to Animals; Exemptions from the Requirement of a Tolerance.* (2022). Available online at: https://www.ecfr.gov/current/title-40/chapter-I/subchapter-E/part-180/subpart-D/section-180.930 (accessed September 30, 2022).

[10] Pest Management Regulatory Agency of Canada. *Proposed Re-Evaluation Decision PRVD2015-01, Glyphosate.* (2015). Available online at: https://www.canada.ca/en/health-canada/services/consumer-product-safety/pesticides-pest-management/public/consultations/proposed-re-evaluation-decisions/2015/glyphosate/document.html (accessed September 30, 2022).

[11] Krogh KA Vejrup KV Mogensen BB Halling-Sørensen B. Liquid chromatography–mass spectrometry method to determine alcohol ethoxylates and alkylamine ethoxylates in soil interstitial water, ground water and surface water samples. *J Chromatogr A.* (2002) 957:45–57. 10.1016/S0021-9673(02)00077-8 12102312

[12] Krogh KA Mogensen BBG Halling-S Rensen B Cort SA Vejrup KV Barcel D. Analysis of alcohol ethoxylates and alkylamine ethoxylates in agricultural soils using pressurised liquid extraction and liquid chromatography?mass spectrometry. *Anal Bioanal Chem.* (2003) 376:1089–97. 10.1007/s00216-003-2062-3 12904945

[13] Wang N Besser JM Buckler DR Honegger JL Ingersoll CG Johnson BT Influence of sediment on the fate and toxicity of a polyethoxylated tallowamine surfactant system (MON 0818) in aquatic microcosms. *Chemosphere.* (2005) 59:545–51. 10.1016/j.chemosphere.2004.12.009 15788177

[14] Corbera M Simonet BM Salvadó V Hidalgo M. Characterisation of alkylamine ethoxylates (ANEOs) in commercial herbicide formulations using liquid chromatography/electrospray ionisation mass spectrometry. *Rapid Commun Mass Spectrom.* (2010) 24:2931–7. 10.1002/rcm.4698 20872624

[15] Tush D Loftin KA Meyer MT. Characterization of polyoxyethylene tallow amine surfactants in technical mixtures and glyphosate formulations using ultra-high performance liquid chromatography and triple quadrupole mass spectrometry. *J Chromatogr A.* (2013) 1319:80–7. 10.1016/j.chroma.2013.10.032 24188997

[16] Tush D Maksimowicz MM Meyer MT. Dissipation of polyoxyethylene tallow amine (POEA) and glyphosate in an agricultural field and their co-occurrence on streambed sediments. *Sci Total Environ.* (2018) 636:212–9. 10.1016/j.scitotenv.2018.04.246 29704716

[17] Rodriguez-Gil JL Lissemore L Solomon K Hanson M. Dissipation of a commercial mixture of polyoxyethylene amine surfactants in aquatic outdoor microcosms: effect of water depth and sediment organic carbon. *Sci Total Environ.* (2016) 550:449–58. 10.1016/j.scitotenv.2016.01.140 26845181

[18] Shao B Hu J Yang M. Determination of nonylphenol ethoxylates in the aquatic environment by normal phase liquid chromatography–electrospray mass spectrometry. *J Chromatogr A.* (2002) 950:167–74. 10.1016/S0021-9673(02)00011-0 11990990

[19] Dufour A Thiébaut D Ligiero L Loriau M Vial J. Chromatographic behavior and characterization of polydisperse surfactants using ultra-high-performance liquid chromatography hyphenated to high-resolution mass spectrometry. *J Chromatogr A.* (2020) 1614:460731. 10.1016/j.chroma.2019.460731 31836311

[20] Jandera P Holčapek M Theodoridis G. Investigation of chromatographic behaviour of ethoxylated alcohol surfactants in normal-phase and reversed-phase systems using high-performance liquid chromatography–mass spectrometry. *J Chromatogr A.* (1998) 813:299–311. 10.1016/S0021-9673(98)00359-8

[21] Lara-Martín PA Gómez-Parra A González-Mazo E. Development of a method for the simultaneous analysis of anionic and non-ionic surfactants and their carboxylated metabolites in environmental samples by mixed-mode liquid chromatography–mass spectrometry. *J Chromatogr A.* (2006) 1137:188–97. 10.1016/j.chroma.2006.10.009 17070820

[22] Liu X Pohl C. New hydrophilic interaction/reversed-phase mixed-mode stationary phase and its application for analysis of nonionic ethoxylated surfactants. *J Chromatogr A.* (2008) 1191:83–9. 10.1016/j.chroma.2007.12.012 18184616

[23] Abrar S Trathnigg B. Separation of nonionic surfactants according to functionality by hydrophilic interaction chromatography and comprehensive two-dimensional liquid chromatography. *J Chromatogr A.* (2010) 1217:8222–9. 10.1016/j.chroma.2010.10.118 21094949

[24] Elsner V Laun S Melchior D Köhler M Schmitz OJ. Analysis of fatty alcohol derivatives with comprehensive two-dimensional liquid chromatography coupled with mass spectrometry. *J Chromatogr A.* (2012) 1268:22–8. 10.1016/j.chroma.2012.09.072 23116798

[25] Hammer J Haftka JJH Scherpenisse P Hermens JLM de Voogt P. Investigating hydrophilic and electrostatic properties of surfactants using retention on two mixed-mode liquid chromatographic columns. *J Chromatogr A.* (2018) 1571:185–92. 10.1016/j.chroma.2018.08.024 30146378

[26] Farsang E Gaál V Horváth O Bárdos E Horváth K. Analysis of non-ionic surfactant triton x-100 using hydrophilic interaction liquid chromatography and mass spectrometry. *Molecules.* (2019) 24:1223. 10.3390/molecules24071223 30925777 PMC6480021

[27] Ferguson PL Iden CR Brownawell BJ. Analysis of nonylphenol and nonylphenol ethoxylates in environmental samples by mixed-mode high-performance liquid chromatography–electrospray mass spectrometry. *J Chromatogr A.* (2001) 938:79–91. 10.1016/S0021-9673(01)01091-3 11771849

[28] Jiang Z Cao X Li H Zhang C Abd El-Aty AM Jin F Fast determination of alkylphenol ethoxylates in leafy vegetables using a modified quick, easy, cheap, effective, rugged, and safe method and ultra-high performance supercritical fluid chromatography–tandem mass spectrometry. *J Chromatogr A.* (2017) 1525:161–72. 10.1016/j.chroma.2017.10.035 29056272

[29] Jiang Z Cao X Li H Zhang C Abd El-Aty AM Jeong JH Rapid analysis of tristyrylphenol ethoxylates in cucumber-field system using supercritical fluid chromatography–tandem mass spectrometry. *Food Chem.* (2018) 266:119–25. 10.1016/j.foodchem.2018.05.122 30381166

[30] Gama MR Da Costa Silva RG Collins CH Bottoli CBG. Hydrophilic interaction chromatography. *TrAC Trends Anal Chem.* (2012) 37:48–60. 10.1016/j.trac.2012.03.009

[31] Yoshida T Hamada H Murakawa H Yoshimoto H Tobino T Toda K. Determination of histamine in seafood by hydrophilic interaction chromatography/tandem mass spectrometry. *Anal Sci.* (2012) 28:179–82. 10.2116/analsci.28.179 22322812

[32] Kumar A Heaton JC McCalley DV. Practical investigation of the factors that affect the selectivity in hydrophilic interaction chromatography. *J Chromatogr A.* (2013) 1276:33–46. 10.1016/j.chroma.2012.12.037 23332781

[33] Zhang Q Yang F Ge L Hu Y Xia Z. Recent applications of hydrophilic interaction liquid chromatographyin pharmaceutical analysis. *J Sep Sci.* (2017) 40:49–80. 10.1002/jssc.201600843 27717145

[34] Wan H Sheng Q Zhong H Guo X Fu Q Liu Y Evaluation of a silicon oxynitride hydrophilic interaction liquid chromatography column in saccharide and glycoside separations. *J Sep Sci.* (2015) 40:1271–6. 10.1002/jssc.201401413 25631584

[35] Shen F Xu Y Wang Y Chen J Wang S. Rapid and ultra-trace levels analysis of 33 antibiotics in water by on-line solid-phase extraction with ultra-performance liquid chromatography-tandem mass spectrometry. *J Chromatogr A.* (2022) 1677:463304. 10.1016/j.chroma.2022.463304 35809524

[36] Osburn QW. Analytical method for a cationic fabric softener in waters and wastes. *J Am Oil Chem Soc.* (1982) 59:453–7. 10.1007/BF02634433

[37] Alexandre B Barbara G Laure W Bruno D Adriana G Emmanuelle V. Development of a multiple-class analytical method based on the use of synthetic matrices for the simultaneous determination of commonly used commercial surfactants in wastewater by liquid chromatography-tandem mass spectrometry. *J Chromatogr A.* (2016) 1450:64–75. 10.1016/j.chroma.2016.04.078 27156752

[38] Ko A Abd El-Aty AM Rahman MM Jang J Kim S Choi J A modified QuEChERS method for simultaneous determination of flonicamid and its metabolites in paprika using tandem mass spectrometry. *Food Chem.* (2014) 157:413–20. 10.1016/j.foodchem.2014.02.038 24679799

[39] Lehotay SJ. QuEChERS sample preparation approach for mass spectrometric analysis of pesticide residues in foods. *Methods Mol Biol.* (2011) 747:65–91. 10.1007/978-1-61779-136-9_4 21643905

[40] Zhu Y Liu X Xu J Dong F Liang X Li M Simultaneous determination of spirotetramat and its four metabolites in fruits and vegetables using a modified quick, easy, cheap, effective, rugged, and safe method and liquid chromatography/tandem mass spectrometry. *J Chromatogr A.* (2013) 1299:71–7. 10.1016/j.chroma.2013.05.049 23768534

[41] Shi X Jin F Huang Y Du X Li C Wang M Simultaneous determination of five plant growth regulators in fruits by modified quick, easy, cheap, effective, rugged, and safe (QuEChERS) extraction and liquid chromatography–tandem mass spectrometry. *J Agr Food Chem.* (2012) 60:60–5. 10.1021/jf204183d 22148585

[42] Wabaidur SM AlAmmari A Aqel A Al-Tamrah SA Alothman ZA Ahmed AYBH. Determination of free fatty acids in olive oils by UPHLC–MS. *J Chromatogr B.* (2016) 1031:109–15. 10.1016/j.jchromb.2016.07.040 27474779

[43] AlFaris NA Wabaidur SM Alothman ZA Altamimi JZ Aldayel TS. Fast and efficient immunoaffinity column cleanup and liquid chromatography–tandem mass spectrometry method for the quantitative analysis of aflatoxins in baby food and feeds. *J Sep Sci.* (2020) 43:2079–87. 10.1002/jssc.20190130745 32125088

[44] Abdullah AlFaris N Zaidan ALTamimi J ALOthman ZA Wabaidur SM Ghafar AA Saleh Aldayel T. Development of a sensitive liquid-liquid extraction and ultra-performance liquid chromatography-tandem mass spectrometry method for the analysis of carbaryl residues in fresh vegetables sold in Riyadh. *J King Saud Univ Sci.* (2020) 32:2414–8. 10.1016/j.jksus.2020.03.030

[45] Schreuder RH Martijn A. Determination of the composition of ethoxylated alkylamines in pesticide formulations by high-performance liquid chromatography using ion-pair extraction detection. *J Chromatogr A.* (1986) 368:339–50.

[46] La Guardia MJ Hale RC Harvey E Mainor TM. Alkylphenol ethoxylate degradation products in Land-Applied sewage sludge (Biosolids). *Environ Sci Technol.* (2001) 35:4798–804. 10.1021/es0109040 11775155

[47] Ferguson PL Bopp RF Chillrud SN Aller RC Brownawell BJ. Biogeochemistry of nonylphenol ethoxylates in urban estuarine sediments. *Environ Sci Technol.* (2003) 37:3499–506. 10.1021/es026335t 12953858

