# Supplementary material for: High-throughput analysis of polyethoxylated tallow amine homologs in citrus using a modified QuEChERS-HILIC-MS method
Source: Front Nutr. 2022 Nov 30;9:1061195. doi: 10.3389/fnut.2022.1061195 (PMC9748182; doi:10.3389/fnut.2022.1061195)
Supplement: Supplementary file 1 [file Table_1.DOCX]

Supplementary Material

# Supplementary Figures and Tables

## Supplementary Figures


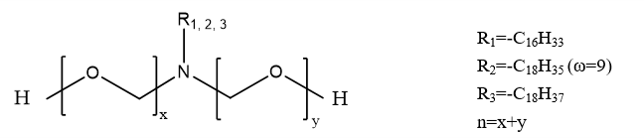


**Supplementary Figure 1.** Chemical structure of synthetic POE-tallowamine. (R is mainly derived from palmitic acid (C16:0), oleic acid (C18:1, ω-9) and stearic acid (C18:0), n means ethoxylate unites (EO), varying from 2 to 23.


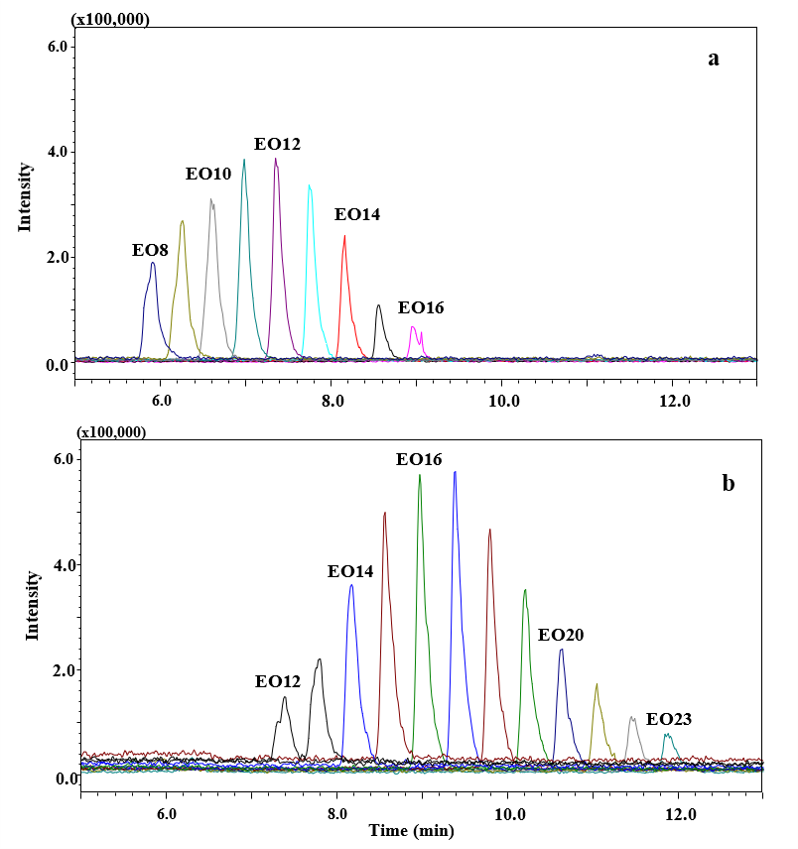


**Supplementary Figure 2.** Chromatograms of single- and double-charged adducts extracted in standard mixtures POE-Tallowamine. a: [M+H]^+^ adducts of C16sEO8-16, b: [M+H+NH_4_]^2+^ adducts of C16sEO12-23.


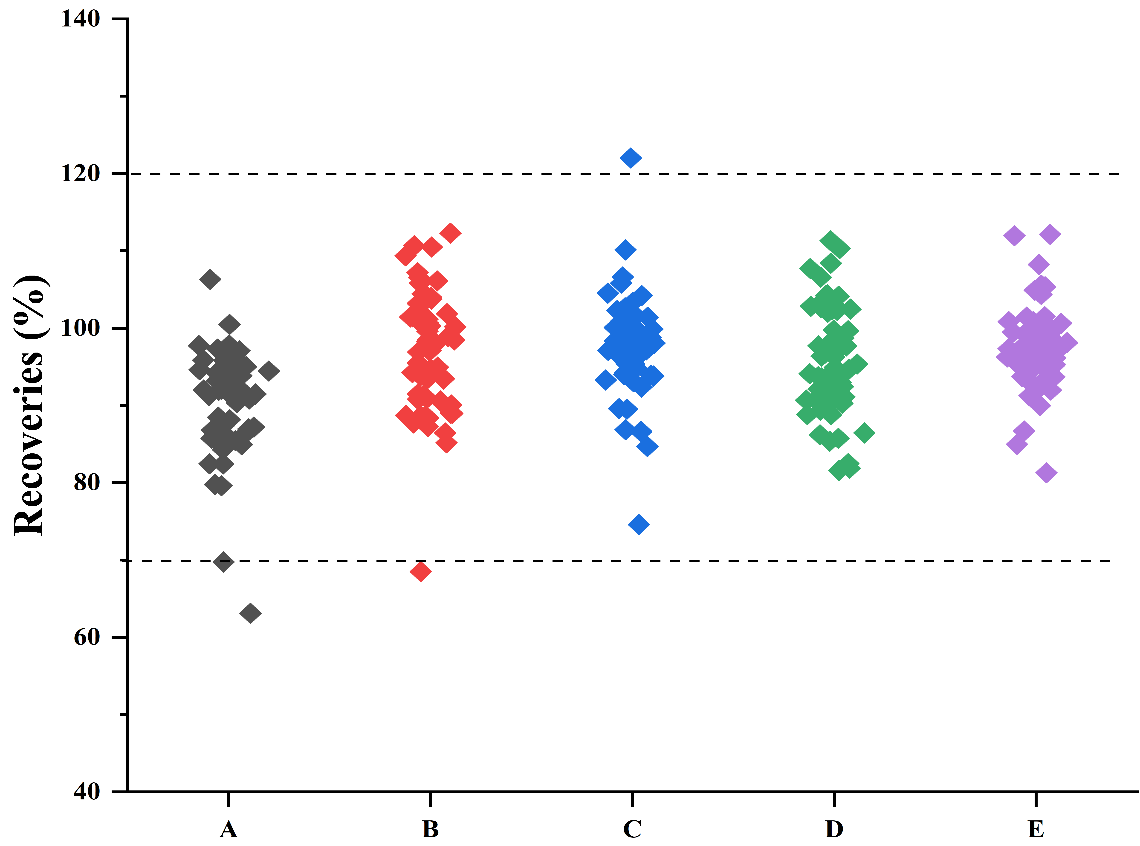


**Supplementary Figure 3.** Recoveries of different [extraction](javascript:;) [solvent](javascript:;)s for POE-Tallowamine homologs in the citrus matrix (n = 3). The peak numbers are the same as in Table S1. Solvent A: acetonitrile; B: 0.1% formic acid in acetonitrile; C: 0.5% formic acid in acetonitrile; D: 1% formic acid in acetonitrile; E: 1.5% formic acid in acetonitrile.


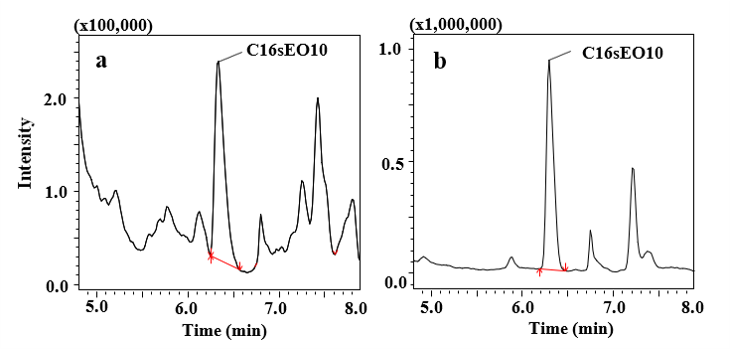


**Supplementary Figure 4.** Cleanup performance of different sorbents. a: 50 mg C18; b: 50 mg PSA.


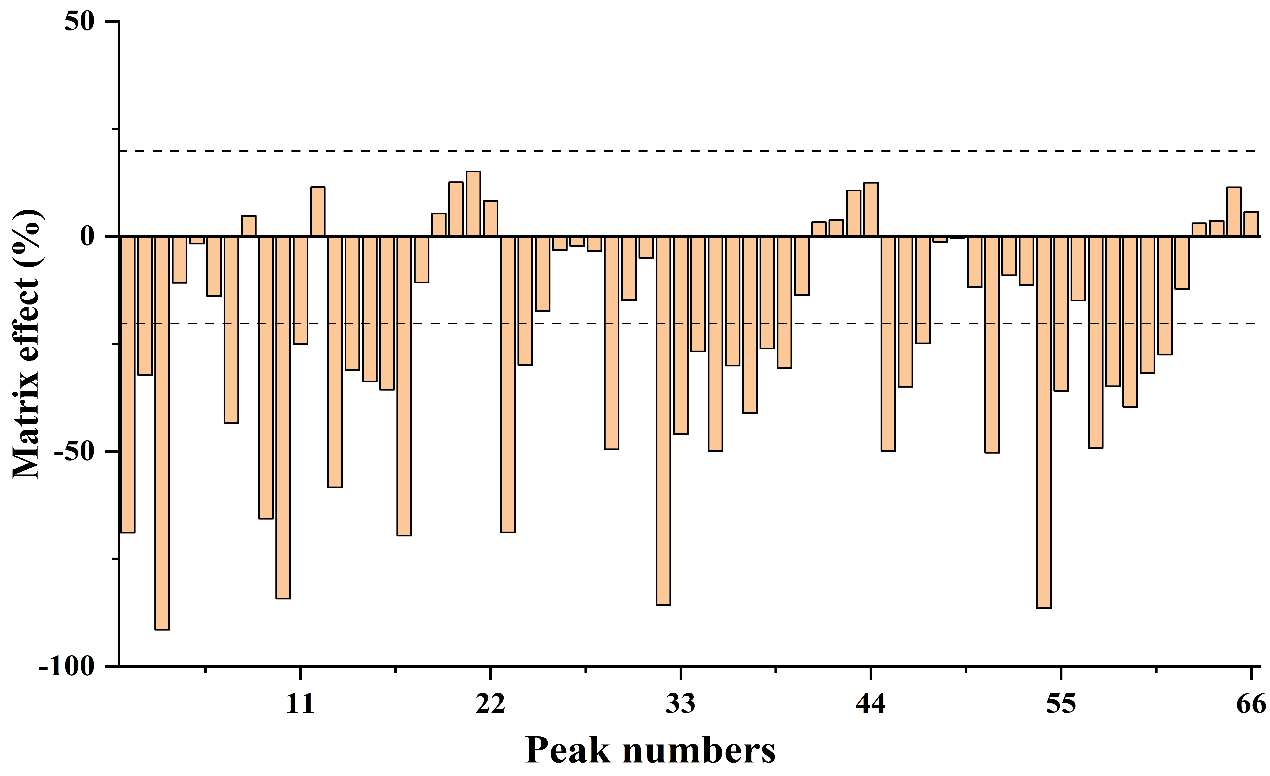
**Supplementary Figure 5.** Matrix effects (MEs) of POE-Tallowamine homologs in citrus. The peak numbers are the same as in Table S1.


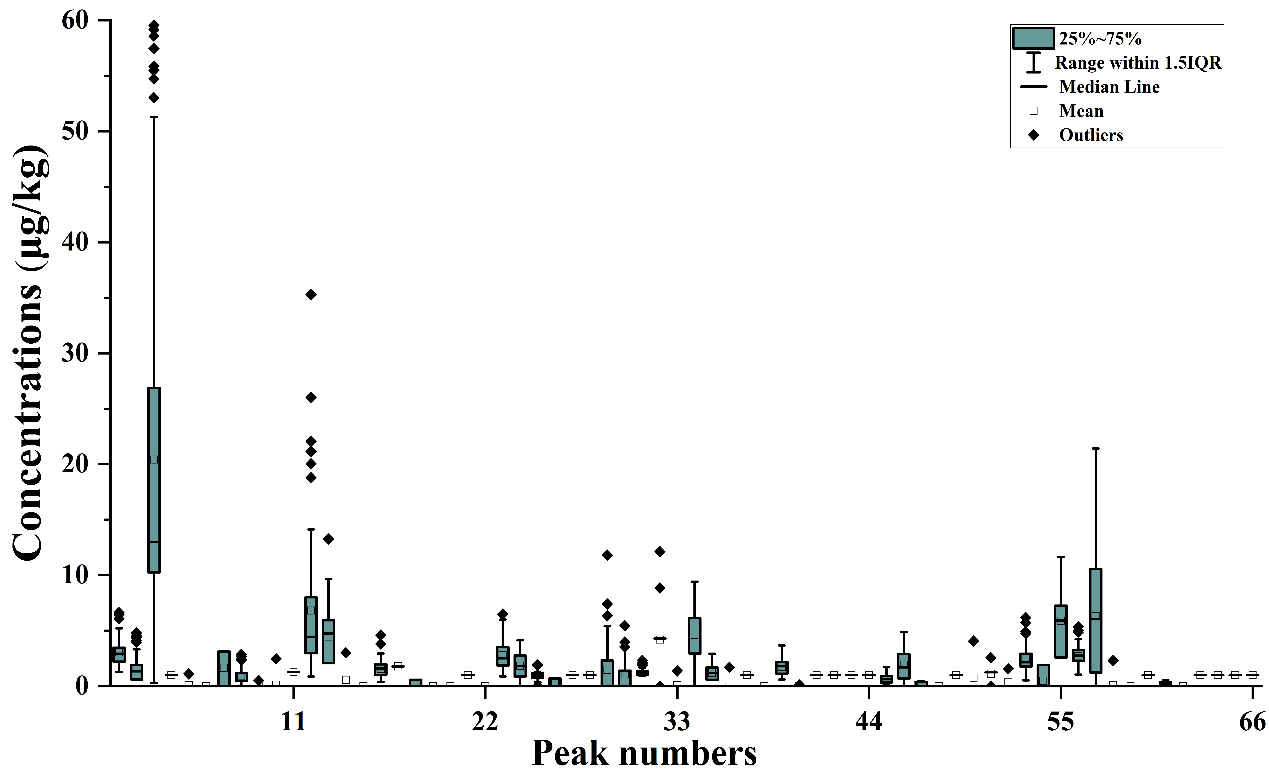


**Supplementary Figure 6.** Concentration profiles of individual POE-Tallowamine homologs in citrus samples. The peak numbers are the same as in Table S1.

## Supplementary Tables

**Table S1 Mass spectrometry conditions of POE-tallowamine (n=2–23).**

| No. | Abbr. Name | RT (min) | Adduct ion | Quantification ion |
| --- | --- | --- | --- | --- |
| 1 | C16s-EO2 | 3.74 | [M+H]^+^ | 330.5 |
| 2 | C16s-EO3 | 4.03 | [M+H]^+^ | 374.2 |
| 3 | C16s-EO4 | 4.29 | [M+H]^+^ | 418.4 |
| 4 | C16s-EO5 | 4.63 | [M+H]^+^ | 462.5 |
| 5 | C16s-EO6 | 4.97 | [M+H]^+^ | 506.6 |
| 6 | C16s-EO7 | 5.33 | [M+H]^+^ | 550.5 |
| 7 | C16s-EO8 | 5.66 | [M+H]^+^ | 594.5 |
| 8 | C16s-EO9 | 5.97 | [M+H]^+^ | 638.6 |
| 9 | C16s-EO10 | 6.37 | [M+H]^+^ | 682.8 |
| 10 | C16s-EO11 | 6.73 | [M+H]^+^ | 726.5 |
| 11 | C16s-EO12 | 7.12 | [M+H]^+^ | 770.7 |
| 12 | C16s-EO13 | 7.52 | [M+H]^+^ | 814.7 |
| 13 | C16s-EO14 | 7.93 | [M+NH_4_+H]^2+^ | 438.4 |
| 14 | C16s-EO15 | 8.41 | [M+NH_4_+H]^2+^ | 460.4 |
| 15 | C16s-EO16 | 8.77 | [M+NH_4_+H]^2+^ | 482.4 |
| 16 | C16s-EO17 | 9.19 | [M+NH_4_+H]^2+^ | 504.4 |
| 17 | C16s-EO18 | 9.64 | [M+NH_4_+H]^2+^ | 526.4 |
| 18 | C16s-EO19 | 10.06 | [M+NH_4_+H]^2+^ | 548.4 |
| 19 | C16s-EO20 | 10.47 | [M+NH_4_+H]^2+^ | 570.5 |
| 20 | C16s-EO21 | 10.87 | [M+NH_4_+H]^2+^ | 592.5 |
| 21 | C16s-EO22 | 11.29 | [M+NH_4_+H]^2+^ | 614.5 |
| 22 | C16s-EO23 | 11.69 | [M+NH_4_+H]^2+^ | 636.7 |
| 23 | C18s-EO2 | 3.64 | [M+H]^+^ | 358.6 |
| 24 | C18s-EO3 | 3.94 | [M+H]^+^ | 402.5 |
| 25 | C18s-EO4 | 4.16 | [M+H]^+^ | 446.4 |
| 26 | C18s-EO5 | 4.50 | [M+H]^+^ | 490.5 |
| 27 | C18s-EO6 | 4.86 | [M+H]^+^ | 534.7 |
| 28 | C18s-EO7 | 5.17 | [M+H]^+^ | 578.5 |
| 29 | C18s-EO8 | 5.52 | [M+H]^+^ | 622.9 |
| 30 | C18s-EO9 | 5.84 | [M+H]^+^ | 666.6 |
| 31 | C18s-EO10 | 6.20 | [M+H]^+^ | 710.7 |
| 32 | C18s-EO11 | 6.55 | [M+H]^+^ | 754.9 |
| 33 | C18s-EO12 | 6.95 | [M+H]^+^ | 798.5 |
| 34 | C18s-EO13 | 7.31 | [M+H]^+^ | 842.9 |
| 35 | C18s-EO14 | 7.72 | [M+NH_4_+H]^2+^ | 452.5 |
| 36 | C18s-EO15 | 8.16 | [M+NH_4_+H]^2+^ | 474.5 |
| 37 | C18s-EO16 | 8.59 | [M+NH_4_+H]^2+^ | 496.5 |
| 38 | C18s-EO17 | 8.98 | [M+NH_4_+H]^2+^ | 518.6 |
| 39 | C18s-EO18 | 9.39 | [M+NH_4_+H]^2+^ | 540.6 |
| 40 | C18s-EO19 | 9.82 | [M+NH_4_+H]^2+^ | 562.6 |
| 41 | C18s-EO20 | 10.20 | [M+NH_4_+H]^2+^ | 584.7 |
| 42 | C18s-EO21 | 10.64 | [M+NH_4_+H]^2+^ | 606.7 |
| 43 | C18s-EO22 | 11.02 | [M+NH_4_+H]^2+^ | 628.7 |
| 44 | C18s-EO23 | 11.43 | [M+NH_4_+H]^2+^ | 650.7 |
| 45 | C18u-EO2 | 3.63 | [M+H]^+^ | 356.4 |
| 46 | C18u-EO3 | 3.94 | [M+H]^+^ | 400.4 |
| 47 | C18u-EO4 | 4.17 | [M+H]^+^ | 444.3 |
| 48 | C18u-EO5 | 4.53 | [M+H]^+^ | 488.5 |
| 49 | C18u-EO6 | 4.86 | [M+H]^+^ | 532.6 |
| 50 | C18u-EO7 | 5.20 | [M+H]^+^ | 576.5 |
| 51 | C18u-EO8 | 5.54 | [M+H]^+^ | 620.6 |
| 52 | C18u-EO9 | 5.85 | [M+H]^+^ | 664.4 |
| 53 | C18u-EO10 | 6.22 | [M+H]^+^ | 708.8 |
| 54 | C18u-EO11 | 6.59 | [M+H]^+^ | 752.8 |
| 55 | C18u-EO12 | 6.95 | [M+H]^+^ | 797.0 |
| 56 | C18u-EO13 | 7.32 | [M+H]^+^ | 840.9 |
| 57 | C18u-EO14 | 7.75 | [M+NH_4_+H]^2+^ | 451.2 |
| 58 | C18u-EO15 | 8.20 | [M+NH_4_+H]^2+^ | 473.5 |
| 59 | C18u-EO16 | 8.63 | [M+NH_4_+H]^2+^ | 495.5 |
| 60 | C18u-EO17 | 8.99 | [M+NH_4_+H]^2+^ | 517.6 |
| 61 | C18u-EO18 | 9.45 | [M+NH_4_+H]^2+^ | 539.6 |
| 62 | C18u-EO19 | 9.87 | [M+NH_4_+H]^2+^ | 561.6 |
| 63 | C18u-EO20 | 10.27 | [M+NH_4_+H]^2+^ | 583.7 |
| 64 | C18u-EO21 | 10.66 | [M+NH_4_+H]^2+^ | 605.7 |
| 65 | C18u-EO22 | 11.09 | [M+NH_4_+H]^2+^ | 627.7 |
| 66 | C18u-EO23 | 11.47 | [M+NH_4_+H]^2+^ | 649.7 |
